# Diagnostic yield and copy number variants findings in 219 adult patients with developmental and epileptic encephalopathy

**DOI:** 10.1002/epi.70360

**Published:** 2026-07-04

**Authors:** Laura Licchetta, Giulia Bruschi, Tania Giangregorio, Carlotta Stipa, Elisa Mannini, Raffaella Minardi, Barbara Mostacci, Valentina Tontini, Tommaso Pippucci, Francesca Bisulli, Pamela Magini

**Affiliations:** ^1^ IRCCS Istituto Delle Scienze Neurologiche di Bologna, full member of European Reference Network EpiCARE Bologna Italy; ^2^ Department of Medical and Surgical Sciences University of Bologna Bologna Italy; ^3^ Department of Biomedical and Neuromotor Sciences University of Bologna Bologna Italy; ^4^ Medical Genetics Unit IRCCS Azienda Ospedaliero‐Universitaria di Bologna Bologna Italy

**Keywords:** adults, array CGH, developmental and epileptic encephalopathies, microarray, SNP array

## Abstract

In a clinical setting, exome sequencing (ES) with copy number variant (CNV) analysis is currently the most effective approach for developmental and epileptic encephalopathies (DEE). However, trio‐based ES is often not feasible in adults, its costs remain prohibitive in certain health care settings, and computational tools for CNV calling still lack sufficient accuracy. Chromosomal microarray (CMA) is indicated as a first‐tier test for CNV detection in patients with DEE, dysmorphisms, and comorbidities. We investigate the role of CNVs in the etiology of DEE in an adult cohort, to define the most appropriate diagnostic approach in this population. A total of 219 patients (male/female: 104/115) with undiagnosed DEE underwent array‐based comparative genomic hybridization/single nucleotide polymorphism arrays as adults. Causative CNVs were identified in 18 patients (8.2%); 14 were deletions (mean size ≈ 2.96 Mb), and four were duplications/triplications (mean size ≈ 3.63 Mb). Thirteen were responsible for known deletion/microduplication syndromes, and four were deletions involving haploinsufficient genes associated with epilepsy/neurodevelopmental disorders. For one deletion, population evidence, reports with overlapping deletions, and clinical databases support a likely pathogenic role. The mean age at CMA diagnosis was 32.4 ± 13 years. An additional 46 patients (21%) then received a molecular diagnosis through alternative approaches. Despite a diagnostic delay of 24.2 ± 14.5 years, CMA enabled a genetic diagnosis in 8.2% of our adult DEE patients, especially in those with dysmorphisms. The diagnostic yield rises to 10.4% when excluding patients diagnosed using other methods. A total of 66.7% of solved cases had direct implications from the diagnosis, supporting the clinical utility of CNV analysis inclusion in the diagnostic workflow for adult patients with DEE.

**Key points**

- CMA identified causative CNVs in 8.2% of adults with undiagnosed DEE.
- The diagnostic yield rises to 10.4% when excluding patients diagnosed in parallel using other approaches.
- Most pathogenic CNVs were de novo and involved known syndromic or epilepsy‐related genes.
- Despite a mean diagnostic delay of 24 years, 66.7% of solved cases had clinical implications for surveillance, management, or counseling.
- CNV analysis remains clinically useful in adult DEE, especially in patients with dysmorphic features.

## 1 INTRODUCTION

Developmental and/or epileptic encephalopathies (DEE) comprise a heterogeneous group of severe neurodevelopmental disorders (NDDs) characterized by early onset, often refractory epileptic seizures, and electroencephalographic abnormalities in the context of developmental impairment, which may further deteriorate as a consequence of ongoing epileptic activity.^1^ Most affected patients are at risk of intellectual disability (ID) and psychiatric/behavioral comorbidities, with an overall poor prognosis.^2^

The advent of next generation sequencing (NGS) has led to remarkable progress in gene discovery and a rapid increase in our understanding of the genetic basis of DEE. More than 900 genes are currently implicated as monogenic causes of DEE, among more than 1000 genes associated with monogenic epilepsies (http://github.com/bahlolab/genes4epilepsy). Most monogenic DEE are caused by de novo pathogenic single nucleotide variants,^3^ each accounting for a small proportion of cases, with considerable genetic heterogeneity. Consequently, exome sequencing (ES) is now considered one of the most effective approaches for DEE, allowing identification of a pathogenic variant in up to 50% of cases.^4^, ^5^ Nevertheless, chromosomal microarray analysis (CMA) enables the detection of pathogenic copy number variants (CNVs) in 8%–16% of cases when epilepsy co‐occurs with NDDs^6^, ^7^ and is regarded as a valuable genetic test for patients presenting with epilepsy and ID and/or dysmorphic features.^8^

Most studies on CNVs in DEE have focused on children, resulting in a large number of published pediatric cohorts. Adults with undiagnosed DEE, often improperly investigated because the disease onset was prior to the most modern cytomolecular techniques, are rarely tested genetically. Moreover, data on CNV analysis in this population remain scarce and generically include patients with drug‐resistant epilepsy and “comorbidities.”^9^ This gap is critical, given the persistent clinical complexity and diagnostic uncertainty in adulthood, often compounded by incomplete historical data, evolving phenotypes, and subtle dysmorphic features. In this context, reevaluation of adult patients with DEE using contemporary genetic tools is essential to refine prognosis, guide clinical management, and provide genetic counseling. Moreover, establishing a genetic diagnosis can provide long‐awaited answers to patients and families, facilitating access to disease‐specific care and support networks.

We investigated the contribution of CNVs to the etiology of DEE in undiagnosed, adult patients. We aim at describing genetic findings and diagnostic yield, and at evaluating the clinical utility of CNV analysis within this population, to define the most effective diagnostic algorithm.

## 2 MATERIALS AND METHODS

### 2.1 Participants, setting, and eligibility criteria

The study was conducted between 2020 and 2025 at the Adult Tertiary Epilepsy Center of our institute. We retrospectively included all adult patients with undiagnosed DEE, defined according to the International League Against Epilepsy (ILAE) criteria,^1^ who were referred between 2020 and 2023 to the Epilepsy Program of our institution and underwent CMA. All patients underwent a comprehensive electroclinical and neuroradiological evaluation, including the assessment of dysmorphic features that were compared between the two groups (patients with pathogenic CNVs vs. patients without pathogenic CNVs) by chi‐squared test. Data regarding any other genetic tests carried out were also collected.

### 2.2 Genetic testing and analysis

CMA was conducted prospectively in all patients except 30, who had the analysis performed as adults, before the transition from pediatric care to our adult epilepsy center. CNV analysis was performed using (1) International Standards for Cytogenomic Arrays (ISCA) 8 × 60K array‐based comparative genomic hybridization (array CGH) 8 × 60K platform and Cytogenomics software (Agilent Technologies) and/or (2) whole genome genotyping data from the Epi25 Collaborative generated using Illumina's Infinium GSA‐MD v1 platform (single nucleotide polymorphism [SNP] array).

#### 2.2.1 Array CGH

Array CGH was performed on samples processed at the Medical Genetics Unit of Scientific Institute for Research, Hospitalization, and Health Care (IRCCS) Azienda Ospedaliero‐Universitaria di Bologna (AOUBO) according to the manufacturer's protocols. Briefly, 200 ng of genomic DNA extracted from peripheral blood was enzymatically labeled with cyanine 5‐labeled nucleotides. A sex‐matched reference DNA sample was labeled with cyanine 3. After purification, patient and reference DNA were cohybridized to the SurePrint G3 ISCA 8 × 60K array, with a median probe spacing of 60 kb and a resolution of 120 kb. Following a 24‐h hybridization, slides were washed and scanned. Fluorescence signals were converted into TIFF image files and analyzed using Cytogenomics 5.0 software, which provided quality metrics and calculated log_2_ ratios for each probe. CNVs were called using the ADM‐1 algorithm with a threshold of 6, requiring at least three consecutive probes with concordant log_2_ ratios. Results were visualized graphically based on genomic coordinates and deviation from the disomic line (log_2_ ratio = 0).

#### 2.2.2 SNP array

Whole genome genotyping microarrays were performed through the Illumina Infinium GSA‐MD v1 platform as part of the Epi25 Collaborative project. Samples included in this project were analyzed through both whole exome sequencing and SNP arrays, regardless of any previous genetic test performed. We decided to use genotyping data to compare CNV calls from SNP arrays and array CGH, when both were available, and obtain CNV data for patients who had not yet undergone array CGH. Raw data were processed at the Computational Genomics Unit of IRCCS AOUBO. To infer CNV calls from genotyping data, we designed a portable workflow using the PennCNV algorithm. We filtered out all samples with an SD of log R ratio > .3, B allele frequency drift > .01, waviness factor > .05, and CNV load > 100. CNV calls that spanned <20 markers, were <20 kb in length, had an SNP density < .0001 (number of markers/length of CNV), and had a microdeletion/microduplication frequency > 1% in the entire study sample were excluded. CNV calls were annotated using AnnotSV software, which provides functionally, regulatory, and clinically relevant information for CNV interpretation.

#### 2.2.3 CNV interpretation

Detected variants were compared against multiple databases: Database of Genomic Variants (DGV), DECIPHER (https://decipher.sanger.ac.uk), ClinVar (https://www.ncbi.nlm.nih.gov/clinvar), and a database of human CNVs hosted by IRCCS Oasi Maria SS (Troina, Italy). Information about genomic regions and genes affected by CNVs and known associated disease was retrieved from the UCSC Genome Browser (http://genome.ucsc.edu/), PubMed, and Online Mendelian Inheritance in Man databases.

CNVs were interpreted and classified in accordance with the American College of Medical Genetics and Genomics guidelines.^10^ Segregation analysis was performed when parents' samples were available to determine the inheritance pattern of CNVs, jointly evaluated by neurologists and geneticists to interpret their clinical significance.

## 3 RESULTS

The final cohort included 219 adult patients with DEE (male/female: 104/115, mean age at last evaluation: 39.36 ± 14.35 years). All but three had undergone other genetic tests in addition to CMA, including karyotype analysis, single gene sequencing, or short‐read whole exome/gene panel sequencing.

Array CGH was performed in 35 patients (16%), SNP array in 96 (43.8%), and both analyses (array CGH and SNP array) in 88 patients (40.2%).

The mean age at the CMA was 35.14 ± 15 years (range = 12–61 years). We identified causative CNVs in 18 patients, corresponding to an overall diagnostic yield of 8.2%. An additional 46 patients (21%) in our cohort received a molecular diagnosis through alternative approaches. Excluding these patients from the final analysis results in an overall diagnostic yield of 10.4% (18/173) for CMA.

Variants of uncertain significance were detected in 23 patients (10.5%; Table S1), whereas 178 patients (81.3%) showed no significative findings.

### 3.1 Causative CNV

**FIGURE 1:**
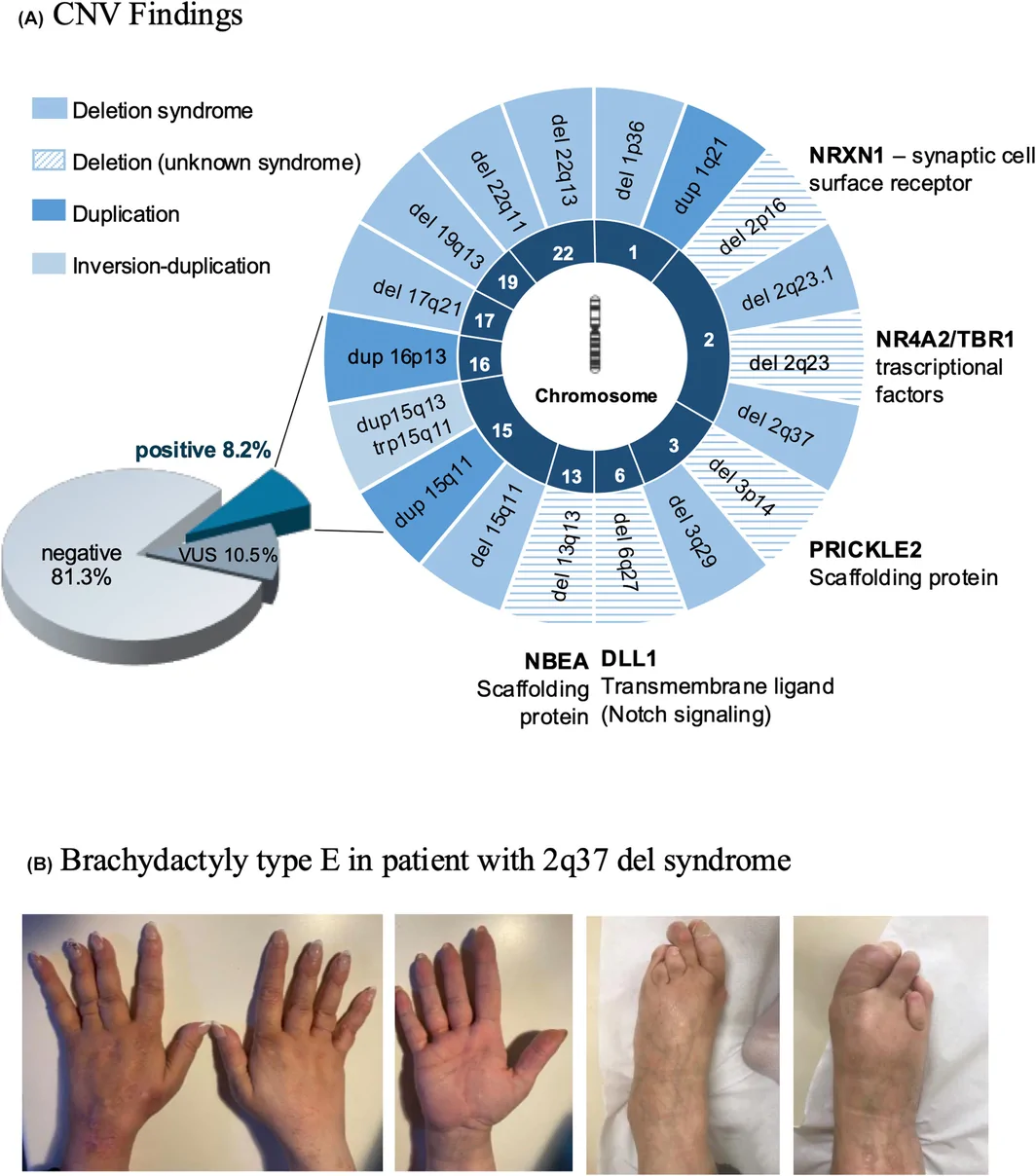
(A) Findings of chromosomal microarray analysis. (B) Brachydactyly type E in Case 6 with 2q37 del syndrome. CNV, copy number variant; VUS, variant of uncertain significance.

Among the 18 pathogenic and likely pathogenic CNV identified (Table 1, Figure 1A), 14 were deletions (mean size ≈ 2.96 Mb) and four were duplications/triplications (mean size ≈ 3.63 Mb). Thirteen CNVs were associated with known deletion/microduplication syndromes in which epilepsy is a recognized clinical feature, whereas four were deletions involving haploinsufficient epilepsy‐related genes (*NRXN1*, *NR4A2*/*TBR1*, *NBEA, DLL1*). Different evidence supported a likely pathogenic classification for the del3p14.2p14.1 in Case 7: the absence of deletions of comparable size in GnomAD and DGV databases, collecting population CNV data; the presence of similar deletions in clinical databases (ClinVar and DECIPHER), classified as pathogenic and reported in patients with complex NDDs; and reports of overlapping de novo deletions in patients with intellectual disability, psychomotor delay, and autistic features.^11^, ^12^, ^13^, ^14^ The deletion includes several protein‐coding genes with a probability of being loss‐of‐function intolerant of 1.00, suggestive of intolerance to loss‐of‐function alterations, including PRICKLE2, a potential candidate in NDDs and epilepsy^15^, ^16^ and included in NGS panels for genetic epilepsy (Genomics England PanelApp). Moreover, short‐read whole exome sequencing performed in Case 7 did not reveal sequence variants responsible for the observed phenotype.

**TABLE 1:** Patients with pathogenic CNVs.

Case #	Sex; age, years	CNV		Inheritance	Known syndrome [# OMIM/ORPHA]	Causative [# OMIM or ClinGen curation ID] or candidate haploinsufficient gene
1	F; 32	del	1p36.33–p36.23	De novo	1p36 deletion syndrome [OMIM 607872]	
2	F; 50	dup	1q21.1–q21.2	Unknown	1q21.1 duplication syndrome [OMIM 612475]	
3	M; 64	del	2p16.3	Unknown	‐	NRXN1 [CCID 007578]
4	F; 17	del	2q23 (mosaic)	De novo	2q23.1 deletion syndrome, MRD1 [OMIM 156200]	
5	F; 37	del	2q23.3–q24.2	De novo	‐	NR4A2 [OMIM 619911–IDLDP], TBR1 [OMIM 606053–IDDAS]
6	F; 42	del	2q37.3	De novo	2q37 deletion syndrome [OMIM 600430]	
7	F; 43	del	3p14.2–p14.1	De novo	‐	PRICKLE2
8	F; 33	del	3q29	De novo	3q29 deletion syndrome [OMIM 609425]	
9	F; 34	del	6q27	De novo	‐	DLL1 [OMIM 618709–NEDBAS]
10	F; 45	del	13q13.2–q13.3	Unknown^a^	‐	NBEA [OMIM 619157*–*NEDEGE]
11	F; 61	del	15q11.2q13.1	Unknown	Angelman syndrome [OMIM 105830]	
12	F; 30	dup	15q11.2–q13.1	De novo	15q11–q13 duplication syndrome [OMIM 608636]	
13	M; 26	trp/dup	15q13.2q13.3 × 3; 15q11.2q13.2 × 4	De novo	Inverted duplicated chromosome 15 syndrome [ORPHA 3306]	
14	M; 49	dup	16p13.1	Maternal	16p13.11 microduplication syndrome [ORPHA 261243]	
15	M; 30	del	17q21.31	De novo	17q21.31 deletion syndrome, Koolen‐De Vries syndrome [OMIM 610443]	
16	F; 26	del	19q12–q13.11	Unknown	19q13.11 deletion syndrome [OMIM 617219]	
17	F; 35	del	22q11.21	Unknown^a^	22q11.2 deletion syndrome [OMIM 188400]	
18	M; 42	del	22q13.33	De novo	22q13.3 deletion syndrome, PHMDS [OMIM 606232]	

Abbreviations: CCID, Coordinating Center for Infectious Diseases; CNV, copy number variant; del, deletion; dup, duplication; F, female; IDDAS, intellectual developmental disorder with autism and speech delay; IDLDP, intellectual developmental disorder with language impairment and early onset dopa‐responsive dystonia‐parkinsonism; M, male; MRD1, autosomal dominant intellectual developmental disorder‐1; NEDBAS, neurodevelopmental disorder with nonspecific brain abnormalities with or without seizures; NEDEGE, neurodevelopmental disorder with or without early onset generalized epilepsy; OMIM, Online Mendelian Inheritance in Man; ORPHA, Orphanet; PHMDS, Phelan–McDermid syndrome; trp, triplication.

^a^ Only mother available for genetic testing.

Segregation analysis, performed in 12 patients, proved that most causative CNVs (67%) occurred de novo.

The subgroup of patients carrying causative CNV included five males and 13 females, with a mean age at last follow‐up of 38.67 ± 11.81 years (range = 17–64 years). The mean age at seizure onset was 9.29 ± 5.04 years (range = 1 month–18 years). The most frequent seizure types were generalized onset (14; 77.8%) and focal onset seizures (11; 61.1%), whereas epileptic spasms occurred in one patient (5.6%). Most patients (11, 61.1%) had drug‐resistant seizures despite previous trials with 1–9 antiseizure medications (mean = 6.28 ± 4.20). Dysmorphic features were observed in 11 (61.1%) cases, proving to be significantly higher compared with patients without pathogenic CNVs (64, 31.8%, *p* = .0121; Figure 1B). Abnormal neurological examination and malformations of cortical development were identified in 15 (83.3%) and six (33.3%) cases, respectively.

Detailed features are in Table S2.

The mean diagnostic delay from disease onset was 24.2 ± 14.5 years (range = 1–53 years).

## 4 DISCUSSION

We investigated the contribution of CNVs to the etiology of DEE in a cohort of 219 adult patients. A causative CNV leading to a genetic diagnosis of DEE was identified in 18 individuals, yielding an overall diagnostic rate of 8.2%.

Direct comparison of our diagnostic rate with previously published data is challenging, as reported rates vary widely depending on the phenotypic homogeneity, genomic testing strategies, laboratory expertise, and rigor of variant interpretation. A meta‐analysis on the diagnostic yield of genetic testing for the epilepsies on mixed‐aged populations with DEE reported an overall diagnostic yield for CGH/CMA of 9%,^5^ similar to our results. Only a few studies have specifically examined CNVs in adults with NDDs and epilepsy, reporting diagnostic yields ranging from 10.8% to 17.7%,^7^, ^9^, ^17^, ^18^, ^19^ with most cohorts clustering around 15%. The lower diagnostic yield observed in our study likely reflects differences in cohort selection. Notably, almost all our patients had undergone further genetic testing in addition to CMA, and 21% were solved by other techniques, thereby reducing the proportion of unresolved cases in which a CNV could be identified. Excluding these cases, the overall yield of CMA increases to 10.4%.

The use of SNP arrays instead of array CGH for part of the cohort could have negatively affected the diagnostic yield. Technical limitations reduce their sensitivity for CNV detection, especially for small imbalances in clinically relevant regions, supporting the diagnostic application of combined CGH + SNP platforms.^20^

Reevaluation of adults with early onset epilepsy diagnosed before the advent of modern genetic testing is essential, particularly for those who underwent limited or now outdated genetic investigations. Despite their current age, this population should still be considered candidates for contemporary genetic testing, in accordance with ILAE recommendations.^19^ Establishing a genetic diagnosis entails substantial benefit for both patients and clinicians in the adult DEE population. It brings closure to the often long and fragmented diagnostic odyssey, reduces unnecessary investigations, avoids inappropriate treatments, and alleviates the emotional and financial burden associated with years of uncertainty. A definitive genetic diagnosis also improves prognostic accuracy, informs eligibility for precision medicine and specific clinical trials, and supports targeted surveillance for syndrome‐specific comorbidities. In our cohort, the most frequently identified pathogenic CNVs were recurrent deletions or duplications at three genomic “hot spots”: 15q11.2, 15q13.2, and 16p13.11.^6^, ^17^, ^21^ In several cases, the detection of a CNV enabled the diagnosis of specific syndromes, such as brachydactyly type E with 2q37 deletion syndrome in Case 6 (Table 1, Figure 1B) and Koolen–De Vries syndrome in Case 15 (Table 1), thereby confirming and refining the clinical suspicions. Despite a diagnostic delay of ~25 years, the newly identified genetic condition in 12 cases (66.7% of solved cases) had direct clinical implications, leading to targeted screening for syndrome‐associated malformations and/or informed therapeutic decisions. Notably, in Case 1 (Table 1), identification of a 1p36 deletion syndrome prompted appropriate cardiological and ophthalmological surveillance. In Case 5 (Table 1), detection of a 2q23.3–q24.2 deletion involving the *NR4A2* gene may guide tailored management of concomitant movement disorders.^22^ In selected cases, genetic findings may also have direct therapeutic implications, for example, by determining eligibility for targeted clinical trials in Angelman syndrome (e.g., GTX‐102).^23^

Beyond these clinical benefits, establishing an etiologic diagnosis enables informed genetic counseling and reproductive planning, while also connecting families to syndrome‐specific organizations and support networks such as the Dup15q Alliance and DEE‐P Connections. These communities provide essential emotional support, shared experiences, and advocacy resources that extend well beyond the scope of medical care.

Although we await a widespread and affordable clinical implementation of long‐read sequencing technologies, short‐read genome sequencing represents the most comprehensive approach for testing DEE patients, enabling the simultaneous detection of single nucleotide variants in coding and noncoding regions and structural variants including CNVs, boosting diagnosis rates in this population.^19^ However, costs remain prohibitive (particularly considering trio‐based analysis), and it implies variant interpretation challenges and high storage data requirements. Accumulating evidence from diagnostic yield and cost‐effectiveness studies supports the use of short‐read ES coupled with CNV analysis as a first‐tier diagnostic strategy for DEE.^24^ However, access to this test through the health care system is not consistent across all Italian regions, and many patients must cover the costs themselves (1200–1800 euros per test). Conversely, array CGH is cheaper (€800–900) and is generally fully covered by the National Health Service (€850). Therefore, CMA offers important advantages in terms of affordability and accessibility, still representing a more economically sustainable first‐line option in selected DEE cases, especially those showing dysmorphic features.^25^ This is supported by the finding in our cohort of a statistically significant higher rate of dysmorphisms in patients carrying pathogenic CNVs compared with those without pathogenic CNVs. In this setting, array CGH may continue to hold a relevant role in reducing disparities in access to genetic testing, particularly in low‐income regions where cost constraints strongly shape diagnostic strategies.

## AUTHOR CONTRIBUTIONS

**Laura Licchetta:** Drafting/revision of the manuscript for content, including medical writing for content; study concept or design. **Giulia Bruschi:** Major role in the acquisition of data; medical writing for content. **Tania Giangregorio:** Genetic analysis and interpretation of data. **Carlotta Stipa:** Major role in the acquisition of data. **Elisa Mannini:** Major role in the acquisition of data. **Raffaella Minardi:** Genetic analysis or interpretation of data. **Barbara Mostacci:** Major role in the acquisition of data. **Valentina Tontini:** Major role in the acquisition of data. **Tommaso Pippucci:** Genetic analysis and interpretation of data. **Francesca Bisulli:** Major role in the acquisition of data. **Pamela Magini:** Drafting/revision of the manuscript for content, including medical writing for content; major role in the acquisition of data.

## FUNDING INFORMATION

The study has been conducted as part of the Multidisciplinary Transition Model for Patients with Complex Epilepsy (TRANS‐EPIC) project, supported by Ricerca Corrente funding from the Italian Ministry of Health.

## CONFLICT OF INTEREST STATEMENT

The author declares no conflicts of interest.

## CONSENT

All participants provided written informed consent for the collection and use of their clinical data for research purposes.

## ETHICAL PUBLICATION STATEMENT

We confirm that we have read the Journal's position on issues involved in ethical publication and affirm that this report is consistent with those guidelines.

## CLINICAL TRIAL REGISTRATION

This study is not a pharmacological clinical trial and has not been registered in a clinical trial registry, as it involves observational assessment of diagnostic methods.

## Supporting information


**TABLE S1.** Variants of uncertain significance were detected in our population.
**TABLE S2.** Clinical features of patients with pathogenic copy number variants.

## Data Availability

The data that support the findings of this study are available on request from the corresponding author. The data are not publicly available due to privacy or ethical restrictions.
