# Supplementary material for: Diagnostic yield and copy number variants findings in 219 adult patients with developmental and epileptic encephalopathy
Source: Epilepsia. 2026 Jul 4;67(9):e141–9. doi: 10.1002/epi.70360 (PMC13592487; doi:10.1002/epi.70360)
Supplement: Supplementary file 1 [file EPI-67-e141-s001.docx]

**Supplemental Table 1**: Variants of uncertain significance were detected in our population.

| Case # | Sex,  Age (y) | CNV | | Inheritance | Candidate epilepsy-related gene  [# OMIM] |
| --- | --- | --- | --- | --- | --- |
| 1 | M, 44 | dup | 1 p22.1 | unknown | KIAA1107 e GLMN |
| 2 | M, 73 | del | 2 q13;  10 q23.1 | unknown | NPHP1 e MTLN  CCSER2 |
| 3 | M, 41 | dup | 3 q27.2;  15 q24.3-q24.2 | paternal  maternal |  |
| 4 | M, 56 | dup | 4 q35.1  4 q35.2 | unknown |  |
| 5 | F, 44 | trp | 5 p14.1-p13.3 | paternal |  |
| 6 | F, 30 | dup | 7 p21.1 | unknown | AGRH, AHR) e 1 gene HGNC |
| 7 | F, 57 | dup | 7 p22.2 | unknown | SDK1 |
| 8 | F, 37 | trip | 7 q36.1  7 q36.1 | maternal |  |
| 9 | F, 29 | dup | 8 p12 | unknown | RBPMS, GTF2E2, SMIM18, GSR e UBXN8 |
| 10 | F, 57 | dup | 8 q21.2 | unknown |  |
| 11 | F, 56 | dup | 10 q22.3 | unknown |  |
| 12 | M, 53 | dup | 10 q25.3 | unknown | HSPA12A MIR9851 ENO4 |
| 13 | M, 50 | del | 13 q33.3 | not maternal |  |
| 14 | F, 28 | dup | 15 q13.3 | unknown |  |
| 15 | F, 36 | del | 15 q15.3 | unknown |  |
| 16 | M, 44 | dup | 15 q25.2 | unknown | AP3B2, WHAMM, HOMER2 |
| 17 | F, 32 | dup | 16p12.3  Xp22.33 | maternal |  |
| 18 | M, 38 | dup | 16q23.2 | maternal |  |
| 19 | M, 54 | dup | 17 p13.2 | paternal |  |
| 20 | M, 37 | dup | 17q25.1  22q11.23 |  |  |
| 21 | F, 46 | dup | 20 q13.13-q13.2 | unknown |  |
| 22 | M,.47 | dup | 21 q11.2 | maternal |  |
| 23 | F, 34 | dup | 21 q11.2 | paternal |  |

**Supplemental Table 2**: Clinical features of patients with pathogenic CNVs.

| Clinical features | | | number | % |
| --- | --- | --- | --- | --- |
| **Age at seizures onset** | | 9.29 ± 5.04 y | | |
|  | < 12 years | | 12 | 66.7 |
|  | 12 -18 years | | 6 | 33.3 |
| **Seizure-semiology** | Spasms | | 1 | 5.6 |
|  | Generalized onset | | 14 | 77.8 |
|  | Focal onset | | 11 | 61.1 |
| **Developmental delay/ regression** |  | | 11 | 61.1 |
| **Intellectual disability** | Mild/moderate | | 11 | 61.1 |
|  | Severe/Profond | | 7 | 38.9 |
| **Psychiatric/behaviour disorders** | | | 14 | 77.8 |
| **Abnormal neurological examination** | | | 15 | 83.3 |
| **Dysmorphic features** | | | 11 | 61.1 |
| **Interictal epileptiform EEG** |  | | 9 | 50.0 |
| **Drug-resistant epilepsy** |  | | 11 | 61.1 |
| **Brain MRI** | Abnormal | | 5 | 27.8 |
|  | Aspecific | | 7 | 38.9 |
|  | Normal | | 6 | 33.3 |
